# Validation List no. 223. Valid publication of new names and new combinations effectively published outside the IJSEM

**DOI:** 10.1099/ijsem.0.006699

**Published:** 2025-05-30

**Authors:** Aharon Oren, Markus Göker

**Affiliations:** 1 The Institute of Life Sciences, The Hebrew University of Jerusalem, The Edmond J. Safra Campus, 9190401 Jerusalem, Israel; 2 Leibniz Institute DSMZ – German Collection of Microorganisms and Cell Cultures, Inhoffenstrasse 7B, 38124 Braunschweig, Germany

The purpose of this list is to publish in the *International Journal of Systematic and Evolutionary Microbiology* (*IJSEM*) those new names and new combinations that were effectively published outside the *IJSEM* as set forth in Rule 27 of the International Code of Nomenclature of Prokaryotes (2022 Revision) [1]. Authors and other individuals wishing to have new names and/or combinations included in future lists should send an electronic copy of the published paper to the IJSEM Editorial Office for confirmation that all of the other requirements for valid publication have been met. It is also a requirement of the *IJSEM* and the International Code of Nomenclature of Prokaryotes (ICNP) that authors of new species, new subspecies and new combinations provide evidence that types are deposited in two publicly accessible culture collections in two different countries. It should be noted that the date of valid publication of these new names and combinations is the date of publication of this list, not the date of the original publication of the names and combinations. The authors of the new names and combinations are given below.

Inclusion of a name on these lists validates the publication of the name and thereby makes it available in the nomenclature of prokaryotes. Inclusion of a name on these lists is part of the requirements of valid publication of an effectively published name. Indeed, some of these names may, in time, be considered later synonyms, or it may be proposed to transfer the organisms to another genus, thus necessitating the creation of a new combination. Conversely, it may be proposed to transfer an organism back to an earlier genus and to regard the basonym or an earlier new combination as the correct name instead of a more recent new combination. The ICNP does not decide on such taxonomic opinions.

**Table:** 

Name/author	Proposed as	Nomenclatural type^1^	Priority^2^	Reference
*Aciduricibacillus* Liu *et al*. 2024, 13^3^	gen. nov.	*Aciduricibacillus chroicocephali*	56	[2]
*Aciduricibacillus chroicocephali* Liu *et al*. 2024, 13^3^	sp. nov.	44XB (=KCTC 43631=MCCC 1K08703)	56	[2]
*Actinoalloteichus fjordicus* Nouioui *et al*. 2017, 1712	sp. nov.	ADI 127–7 (=CECT 9355=DSM 46855)	28	[3]
*Aequorivita ciconiae* Han *et al*. 2025, 10^3^	sp. nov.	H23M31 (=JCM 33229=KCTC 62809)	25	[4]
*Agromyces laixinhei* Cheng *et al*. 2021, 473	sp. nov.	HY052 (=CGMCC 1.17175=JCM 33695)	36	[5]
*Albidovulum aestuarii* (Math *et al*. 2013) He *et al*. 2025, 10^3^	comb. nov. [basonym: *Defluviimonas aestuarii* Math *et al*. 2013]	BS14 (=JCM 18630=KACC 16442)	41	[6]
*Albidovulum aquaemixtae* (Jung *et al*. 2014) He *et al*. 2025, 10^3^	comb. nov. [basonym: *Defluviimonas aquaemixtae* Jung *et al*. 2014]	CDM-7 (=CECT 8626=KCTC 42108)	41	[6]
*Albidovulum denitrificans* (Foesel *et al*. 2013) He *et al*. 2025, 10^3^	comb. nov. [basonym: *Defluviimonas denitrificans* Foesel *et al*. 2013]	D9-3 (=ATCC BAA-1447=DSM 18921)	41	[6]
*Albidovulum litorale* He *et al*. 2025, 9^3^	sp. nov.	WL0050 (=GDMCC 1.3084=JCM 35566=MCCC 1K07524)	41	[6]
*Albidovulum marisflavi* He *et al*. 2025, 9^3^	sp. nov.	WL0002 (=GDMCC 1.2437=JCM 34653=MCCC 1K06013)	41	[6]
*Albidovulum salinarum* He *et al*. 2025, 9^3^	sp. nov.	WL0024 (=GDMCC 1.32438=JCM 34656=MCCC 1K06062)	41	[6]
*Albidovulum sediminicola* He *et al*. 2025, 9^3^	sp. nov.	WL0075 (=GDMCC 1.2419=JCM 34660=MCCC 1K06064)	41	[6]
*Albidovulum sediminis* (Liu *et al*. 2023) He *et al*. 2025, 10^3^	comb. nov. [basonym: *Defluviimonas sediminis* Liu *et al*. 2023]	FT324 (=KCTC 92477=MCCC 1K07685)	41	[6]
*Anaeroselena* Prokofeva *et al*. 2025, 10^3^	gen. nov.	*Anaeroselena agilis*	43	[7]
*Anaeroselena agilis* Prokofeva *et al*. 2025, 10^3^	sp. nov.	4137 cl (=KCTC 25383=VKM B-3575)	43	[7]
*Bifidobacterium kimbladii* Modesto *et al*. 2025, 12^3^	sp. nov.	H1HS16N (=CCUG 76695=DSM 115187)	49	[8]
*Blautia arthritidis* Huang *et al*. 2024, 8^3^	sp. nov.	HA0067 (=CGMCC 1.48059=KCTC 25675)	5	[9]
*Blautia flagellata* Huang *et al*. 2024, 8^3^	sp. nov.	HA1512 (=CGMCC 1.48477=KCTC 25718)	5	[9]
*Blautia immobilis* Huang *et al*. 2024, 9^3^	sp. nov.	HA0440 (=CGMCC 1.48213=KCTC 25742)	5	[9]
*Blautia longa* Huang *et al*. 2024, 9^3^	sp. nov.	HA0030 (=CGMCC 1.48026=KCTC 25674)	5	[9]
*Blautia ovalis* Huang *et al*. 2024, 9^3^	sp. nov.	HA0435 (=CGMCC 1.48208=KCTC 25713)	5	[9]
*Butyricimonas recta* Huang *et al*. 2024, 12^3^	sp. nov.	HA1200 (=CGMCC 1.48382=KCTC 25703)	5	[9]
*Chryseobacterium terrae* Kämpfer *et al*. 2025, 4^3^	sp. nov.	ST-37 (=CCM 9260=LMG 32728)	53	[10]
*Cohnella rhizoplanae* Kämpfer *et al*. 2025, 7^3^	sp. nov.	JJ-181 (=CCM 9031=CIP 112018=DSM 110650=LMG 31678)	29	[11]
*Congregibacter brevis* Tak *et al*. 2024, 746	sp. nov.	IMCC45268 (=KCTC 92921=NBRC 116135)^4^	11	[12]
*Congregibacter variabilis* Tak *et al*. 2024, 745	sp. nov.	IMCC43200 (=CCTCC AB 2023139=KCTC 8133=NBRC 116295)^5^	11	[12]
*Coprococcus immobilis* Huang *et al*. 2024, 9^3^	sp. nov.	HA0444 (=CGMCC 1.48217=KCTC 25745)	5	[9]
*Coprococcus mobilis* Huang *et al*. 2024, 9^3^	sp. nov.	HA0524 (=CGMCC 1.48247=KCTC 25783)	5	[9]
*Eisenbergiella longa* Huang *et al*. 2024, 10^3^	sp. nov.	HA0447 (=CGMCC 1.48220=KCTC 25697)	5	[9]
*Enterococcus immobilis* Huang *et al*. 2024, 7^3^	sp. nov.	HA0446 (=CGMCC 1.48219=KCTC 25678)	5	[9]
*Enterovibrio gelatinilyticus* Huang *et al*. 2025, 10^3^	sp. nov.	ZSDZ42 (=KCTC 82886=MCCC 1K06294)	20	[13]
*Enterovibrio qingdaonensis* Huang *et al*. 2025, 9^3^	sp. nov.	ZSDZ35 (=KCTC 82887=MCCC 1K06293)	20	[13]
*Faecalibacillus hominis* Huang *et al*. 2024, 8^3^	sp. nov.	HA0003 (=CGMCC 1.48003=KCTC 25672)	5	[9]
*Finegoldia dalianensis* Li *et al*. 2024, 7^3,6^	sp. nov.	LY240594 (=GDMCC 1.4375=KCTC 25838)	9	[14]
*Flavobacterium plantiphilum* Kämpfer *et al*. 2025, 4^3,7^	sp. nov.	ST-87 (=CIP 112180=DSM 114790=LMG 32757)	53	[10]
*Flavobacterium rhizophilum* Kämpfer *et al*. 2025, 4^3^	sp. nov.	ST-75 (=CIP 112185=DSM 114831=LMG 32758)	53	[10]
*Flavobacterium rhizosphaerae* Kämpfer *et al*. 2025, 4^3^	sp. nov.	ST-119 (=CIP 112181=DSM 114832=LMG 32756)	53	[10]
*Gaopeijia* Ye *et al*. 2024, 14^3^	gen. nov.	*Gaopeijia maritima*	14	[15]
*Gaopeijia maritima* Ye *et al*. 2024, 14^3^	sp. nov.	DH-78 (=KCTC 102002=MCCC 1H01409)	14	[15]
*Gaopeijiaceae* Ye *et al*. 2024, 15^3^	fam. nov.	*Gaopeijia*	14	[15]
*Gaopeijiales* Ye *et al*. 2024, 15^3^	ord. nov.	*Gaopeijia*	14	[15]
*Halalkalicoccaceae* Mao *et al*. 2025, 11^3^	fam. nov.	*Halalkalicoccus*	15	[16]
*Halalkalicoccus ordinarius* Mao *et al*. 2025, 10^3^	sp. nov.	CG83 (=CGMCC 1.17024=JCM 34143)	15	[16]
*Halalkalicoccus salilacus* Mao *et al*. 2025, 10^3^	sp. nov.	FCH27 (=CGMCC 1.62629=JCM 36674=MCCC 4K00130)	15	[16]
*Halobaculum rarum* Zhu *et al*. 2025, 10^3^	sp. nov.	QDC11 (=KCTC 4308=MCCC 4K00127)	48	[17]
*Halobellus marinus* Zhu *et al*. 2025, 9^3^	sp. nov.	DFY28 (=CGMCC 1.17470=JCM 34310)	48	[17]
*Halobellus ordinarius* Zhu *et al*. 2025, 9^3^	sp. nov.	ZY16 (=CGMCC 1.17476=JCM 34311)	48	[17]
*Halobellus rubicundus* Hwang *et al*. 2024, 8^3^	sp. nov.	MBLA0158 (=JCM 36642=KCTC 4318)	8	[18]
*Haloferax litoreum* Cho *et al*. 2021, 2075	sp. nov.	MBLA0076 (=JCM 34169=KCTC 4288)^8^	44	[19]
*Haloferax marinisediminis* Cho *et al*. 2021, 2079	sp. nov.	MBLA0077 (=JCM 34170=KCTC 4289)^9^	44	[19]
*Haloferax marinum* Cho *et al*. 2021, 2079	sp. nov.	MBLA0078 (=JCM 34171=KCTC 4290)^10^	44	[19]
*Halorarum halobium* Zhu *et al*. 2025, 10^3^	sp. nov.	XH14 (=CGMCC 1.17028=JCM 34145)	48	[17]
*Helicobacter gastrocanis* Rimbara *et al*. 2025, 14^3^	sp. nov.	NHP19-003 (=DSM 111619=JCM 39159)	58	[20]
*Hyphococcus aureus* (Wang *et al*. 2019) Ye *et al*. 2025, 9^3^	comb. nov. [basonym: *Marinicaulis aureus* corrig. Wang *et al*. 2019]	HHTR114 (=KCTC 62394=MCCC 1K03481)	6	[21]
*Hyphococcus formosus* Ye *et al*. 2025, 9^3^	sp. nov.	DH-69 (=KCTC 8010=MCCC 1H00436)	6	[21]
*Hyphococcus lacteus* Ye *et al*. 2025, 9^3^	sp. nov.	ECK-19 (=KCTC 8009=MCCC 1H00435)	6	[21]
*Hyphococcus luteus* Ye *et al*. 2025, 9^3^	nom. nov. [basonym: *Marinicaulis flavus* Yu *et al*. 2019]	SY-3–19 (=KCTC 62156=MCCC 1K03432)	6	[21]
*Jirenia* Huang *et al*. 2024, 6^3^	gen. nov.	*Jirenia arthritidis* ^11^	5	[9]
*Jirenia arthritidis* Huang *et al*. 2024, 6^3^	sp. nov.	HA0569 (=CGMCC 1.48290=KCTC 25701)	5	[9]
*Kaarinaea* Saraf *et al*. 2025, 9^3^	gen. nov.	*Kaarinaea lacus*	30	[22]
*Kaarinaea lacus* Saraf *et al*. 2025, 9^3^	sp. nov.	PCC 9237 (=UHCC 0152)	30	[22]
*Lachnospira acetigenes* Huang *et al*. 2024, 10^3^	sp. nov.	HA2201 (=CGMCC 1.48531=KCTC 25788)	5	[9]
*Lachnospira flagellata* Huang *et al*. 2024, 10^3^	sp. nov.	HA1242 (=CGMCC 1.48424=KCTC 25682)	5	[9]
*Lachnospira hominis* Huang *et al*. 2024, 10^3^	sp. nov.	HA1498 (=CGMCC 1.48463=KCTC 25786)	5	[9]
*Lactonifactor hominis* Huang *et al*. 2024, 11^3^	sp. nov.	HA0443 (=CGMCC 1.48216=KCTC 25744)	5	[9]
*Leeuwenhoekiella obamensis* Machii *et al*. 2025, 11^3^	sp. nov.	FRT2 (=BCRC 81451=DSM 118489=JCM 36940)	50	[23]
*Leifsonia stereocauli* Zuo *et al*. 2025, 6^3^	sp. nov.	YIM 134122 (=CGMCC 1.62033=KCTC 59219=MCCC 1K08590)	24	[24]
*Lentisalinibacter* Lian *et al*. 2025, 4^3^	gen. nov.	*Lentisalinibacter sediminis*	39	[25]
*Lentisalinibacter orientalis* Lian *et al*. 2025, 4^3^	sp. nov.	YNA5901 (=KCTC 8014=MCCC 1H00192)	39	[25]
*Lentisalinibacter salinarum* Lian *et al*. 2025, 4^3^	sp. nov.	X77 (=KCTC 8035=MCCC 1H00221)	39	[25]
*Lentisalinibacter sediminis* Lian *et al*. 2025, 4^3^	sp. nov.	C23 (=KCTC 8013=MCCC 1H00409)	39	[25]
*Limnobacter profundi* Kim *et al*. 2024, 11^3^	sp. nov.	SAORIC-580 (=KACC 21400=NBRC 114111)	13	[26]
*Markusia* corrig. Huang *et al*. 2024, 7^3,12^	gen. nov.	*Markusia hominis* corrig.^11^	5	[9]
*Markusia hominis* corrig. Huang *et al*. 2024, 7^3^	sp. nov.	HA1496 (=CGMCC 1.48461=KCTC 25785)	5	[9]
*Methanimicrococcus hacksteinii* Protasov and Brune 2024, 12^3^	sp. nov.	At1 (=DSM 115570=JCM 39383)	7	[27]
*Methanimicrococcus hongohii* Protasov and Brune 2024, 12^3^	sp. nov.	Hf6 (=DSM 114388=JCM 39385)	7	[27]
*Methanimicrococcus stummii* Protasov and Brune 2024, 12^3^	sp. nov.	Es2 (=DSM 114387=JCM 39384)	7	[27]
*Methanolapillus* Protasov and Brune 2024, 11^3^	gen. nov.	*Methanolapillus millepedarum*	7	[27]
*Methanolapillus africanus* Protasov and Brune 2024, 12^3^	sp. nov.	Ag5 (=DSM 115569=JCM 39381)	7	[27]
*Methanolapillus millepedarum* Protasov and Brune 2024, 12^3^	sp. nov.	Ac7 (=DSM 114425=JCM 39380)	7	[27]
*Methanolapillus ohkumae* Protasov and Brune 2024, 12^3^	sp. nov.	Am2 (=DSM 114424=JCM 39382)	7	[27]
*Methanosarcina baikalica* Zhilina *et al*. 2024, 721	sp. nov.	Z-7115 (=JCM 39438=VKM B-3565)	10	[28]
*Methylocystis borbori* Kaparullina *et al*. 2025, 12^3^	sp. nov.	9N (=KCTC 92566=VKM B-3616)	38	[29]
*Moorella carbonis* Böer *et al*. 2024, 12^3,13^	sp. nov.	ACPs (=CCOS 2103=DSM 116161)	54	[30]
*Naizhengia* Huang *et al*. 2024, 5^3^	gen. nov.	*Naizhengia acetigignens* ^11^	5	[9]
*Naizhengia acetigignens* Huang *et al*. 2024, 5^3^	sp. nov.	HA0073 (=CGMCC 1.48065=KCTC 25694)	5	[9]
*Natronorarus* Mao *et al*. 2025, 11^3^	gen. nov.	*Natronorarus salvus*	15	[16]
*Natronorarus salvus* Mao *et al*. 2025, 11^3^	sp. nov.	CGA53 (=CGMCC 1.16848=JCM 34309)	15	[16]
*Natronosporangium* Sorokin *et al*. 2022, 9^3^	gen. nov.	*Natronosporangium hydrolyticum*	27	[31]
*Natronosporangium hydrolyticum* Sorokin *et al*. 2022, 9^3^	sp. nov.	ACPA39 (=DSM 106523=VKM 2772)	27	[31]
*Neobacillus driksii* Hameed *et al*. 2025, 8^3^	sp. nov.	179-C4-2-HS (=DSM 115941=NRRL B65665)	34	[32]
*Neptunitalea lumnitzerae* Sahin *et al*. 2025, 6^3^	sp. nov.	Y10 (=DSM 110280=NBRC 115783)	51	[33]
*Pannonibacter tanglangensis* Wang *et al*. 2024, 735	sp. nov.	XCT-34 (=GDMCC 1.1947=KCTC 82332)	37	[34]
*Parabacteroides propionicigenes* Huang *et al*. 2024, 12^3^	sp. nov.	HA3406 (=CGMCC 1.48595=KCTC 25789)	5	[9]
*Parahalioglobus* Montecillo 2024, 1837	gen. nov.	*Parahalioglobus pacificus*	3	[35]
*Parahalioglobus pacificus* (Park *et al*. 2012) Montecillo 2024, 1837	comb. nov. [basonym: *Halioglobus pacificus* Park *et al*. 2012]	S1-72 (=DSM 27932=KCTC 23430=NBRC 107742)	3	[35]
*Paralabilibaculum* Zhuang *et al*. 2025, 8^3^	gen. nov.	*Paralabilibaculum antarcticum*	22	[36]
*Paralabilibaculum antarcticum* Zhuang *et al*. 2025, 8^3^	sp. nov.	DW002 (=KCTC 25274=MCCC 1K06067)	22	[36]
*Pedobacter soyangensis* Joung *et al*. 2014, 85^14^	sp. nov.	HME6451 (=CECT 7865=KCTC 23467)	47	[37]
*Peptoniphilus hominis* Huang *et al*. 2024, 12^3^	sp. nov.	HA1503 (=CGMCC 1.48468=KCTC 25716)	5	[9]
*Phenylobacterium ferrooxidans* Pouder *et al*. 2025, 12^3^	sp. nov.	HK31-G (=DSM 116432=LMG 33376=UBOCC M-3429)	57	[38]
*Photobacterium pectinilyticum* Ding *et al*. 2024, 8^3^	sp. nov.	ZSDE20 (=KCTC 82885=MCCC 1K06283)	20	[39]
*Photorhabdus viridis* Machado *et al*. 2024, 8^3^	sp. nov.	Green=MJ2C (=CCM 9407=CCOS 2117)	59	[40]
*Pseudoclavibacter albus* (Lin *et al*. 2004) Vandamme *et al*. 2022, 470	comb. nov. [basonym: *Zimmermannella alba* Lin *et al*. 2004]	LMG 31817 (=CCUG 50212=JCM 12865=NBRC 15616)^15^	2	[41]
*Pyramidobacter arthritidis* Huang *et al*. 2024, 13^3^	sp. nov.	HA0566 (=CGMCC 1.48287=KCTC 25698)	5	[9]
*Rhodoligotrophos ferricapiens* Kim *et al*. 2025, 10^3^	sp. nov.	CJ14 (=JCM 36057=KACC 23063)	23	[42]
*Roseateles caseinilyticus* So *et al*. 2024, 7^3^	sp. nov.	P7 (=KACC 22504=TBRC 15694)	18	[43]
*Roseateles cellulosilyticus* So *et al*. 2024, 9^3^	sp. nov.	P8 (=KACC 22505=TBRC 15695)	18	[43]
*Roseateles microcysteis* Kim *et al*. 2025, 7^3^	sp. nov.	MS17 (=KCTC 8001=LMG 33142)	40	[44]
*Roseiconus* Kumar *et al*. 2021, 748	gen. nov.	*Roseiconus nitratireducens*	46	[45]
*Roseiconus lacunae* Kumar *et al*. 2021, 749	sp. nov.	JC635 (=KCTC 72164=NBRC 113875)	46	[45]
*Roseiconus nitratireducens* Kumar *et al*. 2021, 748	sp. nov.	JC645 (=KCTC 72174=NBRC 113879)	46	[45]
*Roseomonas alba* So *et al*. 2023, 1018^16^	sp. nov.	HJA6 (=KACC 21545=NBRC 114316)	17	[46]
*Roseomonas indoligenes* So *et al*. 2023, 1019^17^	sp. nov.	SG15 (=KCTC 82686=NBRC 114481)	17	[46]
*Roseomonas nitratireducens* So *et al*. 2023, 1019^18^	sp. nov.	PWR1 (=KCTC 82687=NBRC 114490)	17	[46]
*Salinarimonas chemoclinalis* Messner *et al*. 2024, 9^3^	sp. nov.	ML10 (=DSM 118510=NCIMB 15586)	1	[47]
*Salinilacustrithrix* corrig. Gao *et al*. 2024, 11^3,19^	gen. nov.	*Salinilacustrithrix flava*	32	[48]
*Salinilacustrithrix flava* Gao *et al*. 2024, 11^3^	sp. nov.	EGI L10123 (=CGMCC 1.19137=KCTC 49680)	32	[48]
*Salinilacustritrichaceae* Gao *et al*. 2024, 11^3,20^	fam. nov.	*Salinilacustrithrix*	32	[48]
*Sanxizhangella* Huang *et al*. 2024, 5^3^	gen. nov.	*Sanxizhangella immobilis* ^11^	5	[9]
*Sanxizhangella immobilis* Huang *et al*. 2024, 5^3^	sp. nov.	HA0013 (=CGMCC 1.48011=KCTC 25673)	5	[9]
*Sinimarinibacterium thermocellulolyticum* Jiang and Li 2025, 13^3^	sp. nov.	HSW-8 (=GDMCC 1.4313=KCTC 92765)	26	[49]
*Skermanella cutis* Choi *et al*. 2025, 15^3^	sp. nov.	TT6 (=JCM 34945=KCTC 82306)	31	[50]
*Sphingomonas plantiphila* Kämpfer *et al*. 2025, 4^3^	sp. nov.	ST-64 (=CCM 9261=CIP 112178=DSM 114515=LMG 32729)	53	[10]
*Streptococcus dentalis* Goo *et al*. 2024, 979	sp. nov.	S1 (=JCM 36526=KCTC 21234)	12	[51]
*Streptococcus gingivalis* Goo *et al*. 2024, 980	sp. nov.	S2 (=JCM 36527=KCTC 21235)	12	[51]
*Streptococcus immobilis* Huang *et al*. 2024, 8^3^	sp. nov.	HA0527 (=CGMCC 1.48250=KCTC 25679)	5	[9]
*Streptococcus lingualis* Goo *et al*. 2024, 981	sp. nov.	S5 (=JCM 36528=KCTC 21236)	12	[51]
*Streptomyces sirii* Zakalyukina *et al*. 2024, 12^3^	sp. nov.	BP-8 (=CCTCC AA 2024094=VKM Ac-3066)	21	[52]
*Streptomyces yaizuensis* Takahashi *et al*. 2025, 41^21^	sp. nov.	YSPA8 (=NBRC 115866=TBRC 17196)	45	[53]
*Thalassotalea aquiviva* Lee *et al*. 2024, 1107	sp. nov.	273M-4 (=KCTC 8644=LMG 33695)	16	[54]
*Thalassotalea maritima* Lee *et al*. 2024, 1109	sp. nov.	Sam97 (=KCTC 8645=LMG 33694)	16	[54]
*Thermodesulfovibrio autotrophicus* Maltseva *et al*. 2024, 10^3^	sp. nov.	3907–1M (=DSM 112797=JCM 39445=UQM 41601=VKM B-3594)	42	[55]
*Thermodesulfovibrio obliviosus* Maltseva *et al*. 2024, 10^3^	sp. nov.	3462–1 (=JCM 39444=UQM 41602=VKM B-3714)	42	[55]
*Trichloromonadaceae* Waite *et al*. 2020, 6002	fam. nov.	*Trichloromonas*	33	[56]
*Trichloromonas* Waite *et al*. 2020, 6001	gen. nov.	*Trichloromonas acetexigens*	33	[56]
*Trichloromonas acetexigens* (Finster *et al*. 1997) Waite *et al*. 2020, 6002	comb. nov. [basonym: *Desulfuromonas acetexigens* Finster *et al*. 1997]	2873 (=ATCC 51529=DSM 1397)	33	[56]
*Veillonella orientalis* Mashima-Usami *et al*. 2025, 6^3^	sp. nov.	S12025-13 (=CCUG 74596=JCM 33967)	4	[57]
*Virgibacillus salidurans* Oh *et al*. 2025, 7^3^	sp. nov.	NKC19-16 (=DSM 112708=KACC 22327)	55	[58]
*Virgibacillus saliphilus* Oh *et al*. 2025, 6^3^	sp. nov.	NKC19-3 (=DSM 112707=KACC 22326)	55	[58]
*Viridibacterium* Kang *et al*. 2017, 517	gen. nov.	*Viridibacterium curvum*	35	[59]
*Viridibacterium curvum* Kang *et al*. 2017, 518	sp. nov.	JJ016 (=JCM 18715=KACC 16899)	35	[59]
*Vreelandella halophila* (Fendrich 1989) de la Haba *et al*. 2025, 1^3,22^	comb. nov. [basonym: *Pseudomonas halophila* Fendrich 1989]	III (=ATCC 49240=CECT 5286=CIP 105504=DSM 3051=JCM 21223=NBRC 102410)^23^	52	[60]
*Yonghella* Huang *et al*. 2024, 5^3^	gen. nov.	*Yonghella fusiformis* ^11^	5	[9]
*Yonghella fusiformis* Huang *et al*. 2024, 5^3^	sp. nov.	HA1168 (=CGMCC 1.48350=KCTC 25681)	5	[9]
*Zhonglingia* Huang *et al*. 2024, 5^3^	gen. nov.	*Zhonglingia intestinalis* ^11^	5	[9]
*Zhonglingia intestinalis* Huang *et al*. 2024, 5^3^	sp. nov.	HA1519 (=CGMCC 1.48484=KCTC 25719)	5	[9]

For references to Validation Lists 1-71, see *Int J Syst Bacteriol* **49** (1999) 1325. Lists 72-197 were published in *Int J Syst Evol Microbiol* **50** (2000) 3, 423, 949, 1415, 1699, 1953; and **51** (2001) 1, 263, 793, 1229, 1619, 1945; and **52** (2002) 3, 685, 1075, 1437, 1915; and **53** (2003) 1, 373, 627, 935, 1219, 1701; and **54** (2004) 1, 307, 631, 1005, 1425, 1909; and **55** (2005) 1, 547, 983, 1395, 1743, 2235; and **56** (2006) 1, 499, 925, 1459, 2025, 2507; and **57** (2007) 1, 433, 893, 1371, 1933, 2449; and **58** (2008) 1, 529, 1057, 1511, 1993, 2471; and **59** (2009) 1, 451, 923, 1555, 2129, 2647; and **60** (2010) 1, 469, 1009, 1477, 1985, 2509; **61** (2011) 1, 475,1011, 1499, 2025, 2563; and **62** (2012) 1, 473, 1017, 1443, 2045, 2549; and **63** (2013) 1, 797, 1577, 2365, 3131, 3931; and **64** (2014) 1, 693, 1455, 2184, 3603; and **65** (2015), 1, 741, 1112, 2017, 2777, 3767; and **66** (2016) 1, 1603, 1913, 2463, 3761, 4299; and **67** (2017) 1, 529, 1095, 2075, 3140, 4291; and **68** (2018), 1, 693, 1411, 2130, 2707, 3379; and **69** (2019), 5, 597, 1247, 1844, 2627, 3313, and **70** (2020) 1, 1443, 2960, 4043, 4844, 5596. Lists 197-222 were published in *Int J Syst Evol Microbiol* **71** (2021) 004600, 004688, 004773, 004846, 004943, 005096; and **72** (2022) 005167, 005260, 005331, 005422, 005517, 005592; and **73** (2023) 005709, 005812, 005845, 005931, 005997, 006080; and **74** (2024) 006173, 006229, 006275, 006398, 006452, 006501; and **75** (2025) 006562, 006640.

1Abbreviations of culture collections cited in this list can be found at https://lpsn.dsmz.de/text/collections.

2Priority number is assigned according to the date the documentation and request for validation are received.

3The journal in which the name was effectively published does not have continuous pages.

4The effective publication states that the type strain was also deposited as HNIBRBA10383, but no documentation was supplied.

5The effective publication states that the type strain was also deposited as HNIBRBA4496, but no documentation was supplied.

6The etymology must state da.lian.en’sis. N.L. fem. adj. *dalianensis*, ….

7The epithet was given as *plantiphilus* in the protologue heading. Syllabification and etymology must be as follows: plan.ti’phi.lum. L. fem. n. *planta*, plant; Gr. masc. adj. *philos*, loving; N.L. neut. adj. *plantiphilum*, plant-loving.

8The JCM number was given as 34,169 instead of 34169.

9The JCM number was given as 34,170 instead of 34170.

10The JCM number was given as 34,171 instead of 34171.

11The authors did not designate a type species for the new genus; the only species described is, therefore, the type species (Rule 20c); no separate protologue was given for the genus.

12The list editors have corrected the genus name to *Markusia* (Mar.ku’si.a. N.L. fem. n. *Markusia*, …).

13The protologue heading must state *Moorella carbonis* instead of *M. carbonis*.

14Syllabification and etymology must be as follows: so.yang.en’sis. N.L. masc. adj. *soyangensis*, of Soyang, Korea…

15The effective publication states that the type strain was also deposited as IAM 14724 and TISTR 1510, but no documentation was supplied.

16In the title of the paper, the abstract and the phylogenetic tree, *Neoroseomonas alba* was given instead of *Roseomonas alba*.

17In the title of the paper, the abstract and the phylogenetic tree, *Paraoseomonas indoligenes* was given instead of *Roseomonas indoligenes*.

18In the title of the paper and the abstract, *Neoroseomonas nitratireducens* was given instead of *Roseomonas nitratireducens*.

19The name was incorrectly spelled *Salinilacustristhrix* in the protologue heading and in the protologue of the family *Salinilacustritrichaceae*.

20The preferred syllabification is Sa.li.ni.la.cus.tri.tri.cha.ce’ae.

21The etymology must state N.L. masc. adj. instead of N.L. masc./fem. adj.

22Replacement name for the illegitimate name *Vreelandella utahensis* (Sorokin and Tindall 2006) de la Haba *et al*. 2024.

23The effective publication states that the type strain was also deposited as IAM 14440, but no documentation was supplied.

**Corrections to the Validation List no. 221** [61]:

Reference 44 should be corrected as follows: **Dong X, Jia H, Yu Y, Xiang Y, Zhang Y**. Correction for Dong *et al*., ‘Genomic revisitation and reclassification of the genus Providencia’. *mSphere* 2024;9:e00288-24.

## Abbreviations

ICNP: International Code of Nomenclature of Prokaryotes
IJSEM: *International Journal of Systematic and Evolutionary Microbiology*
